# The Role of Biomarkers in Adrenocortical Carcinoma: A Review of Current Evidence and Future Perspectives

**DOI:** 10.3390/biomedicines9020174

**Published:** 2021-02-10

**Authors:** Maja Mizdrak, Tina Tičinović Kurir, Joško Božić

**Affiliations:** 1 Department of Nephrology and Hemodialysis, University Hospital of Split, 21000 Split, Croatia; mmizdrak@mefst.hr; 2 Department of Pathophysiology, University of Split School of Medicine, 21000 Split, Croatia; tticinov@mefst.hr; 3 Department of Endocrinology, Diabetes and Metabolic Disorders, University Hospital of Split, 21000 Split, Croatia

**Keywords:** adrenocortical carcinoma, biomarkers, steroidogenesis, pathophysiology, hormones, steroid profiling, microRNA, next-generation sequencing, prognosis, survival

## Abstract

Adrenocortical carcinoma (ACC) is a rare endocrine malignancy arising from the adrenal cortex often with unexpected biological behavior. It can occur at any age, with two peaks of incidence: in the first and between fifth and seventh decades of life. Although ACC are mostly hormonally active, precursors and metabolites, rather than end products of steroidogenesis are produced by dedifferentiated and immature malignant cells. Distinguishing the etiology of adrenal mass, between benign adenomas, which are quite frequent in general population, and malignant carcinomas with dismal prognosis is often unfeasible. Even after pathohistological analysis, diagnosis of adrenocortical carcinomas is not always straightforward and represents a great challenge for experienced and multidisciplinary expert teams. No single imaging method, hormonal work-up or immunohistochemical labelling can definitively prove the diagnosis of ACC. Over several decades’ great efforts have been made in finding novel reliable and available diagnostic and prognostic factors including steroid metabolome profiling or target gene identification. Despite these achievements, the 5-year mortality rate still accounts for approximately 75% to 90%, ACC is frequently diagnosed in advanced stages and therapeutic options are unfortunately limited. Therefore, imperative is to identify new biological markers that can predict patient prognosis and provide new therapeutic options.

## 1. Introduction

Adrenal tumors are common in the general population, with a prevalence of 3% to 10% and the majority of them are small benign non-functional adrenocortical adenomas [1]. On the contrary, primary adrenal malignancies are rare and malignant tumors of the adrenal gland are most commonly metastases from extra-adrenal sites [2]. Adrenocortical carcinoma (ACC) is a rare primary solid malignancy that arises from the adrenal cortex with an estimated incidence of 0.7–2.0 cases/million habitants/year [3,4]. It can occur at any age, with two peaks of incidence: in early childhood and between the fifth and seventh decades of life with a predilection for the female gender (1.5–2.5:1) [1,3,5]. ACCs usually show aggressive biological behavior and in 40%–60% of patients there are symptoms and signs of hormonal hyperproduction [1]. One third of patients presents with nonspecific symptoms due to local tumor growth, such as abdominal fullness, pain, weakness or early satiety [1]. Approximately 20% to 30% of carcinomas are incidentally diagnosed by imaging procedures for unrelated medical issues [1]. Because of poor prognosis for patients who are diagnosed in advanced stages, it is challenging to maintain a high suspicion of malignancy in those to whom adrenal incidentalomas have been diagnosed [2]. Over the last decades, (epi)genetic analyses and genome-wide expression profile studies have offered major advances in the understanding of the molecular genetics of ACC [6]. However, their clinical utility has not been broadly integrated and ACCs still have poor prognosis with a 5-year mortality rate of approximately 75% to 90% [7]. Despite novel discoveries and modern technologies, curative approaches are still limited and the unfavorable outcome has not improved over the past 40 years [2,6]. By the time of diagnosis, most patients have loco-regional or distant advanced disease often with hormonal hypersecretion that increases morbidity or develop local recurrence and distant metastasis after surgical treatment [5,6]. So far, studies with large cohorts of ACC patients were missing because of the rarity of the disease; however, recent international efforts provided new insights in pathophysiology and treatment [8]. The ACC work-up requires a holistic multidisciplinary expert approach to a single patient since the diagnosis of ACC is not always obvious and represents a challenging task with the possibility of making severe mistakes. The aim of this review was to summarize well established and experimental biomarkers of adrenocortical cancer, including biochemical, pathohistological and molecular aspects of diseases, to analyze their utility in everyday clinical diagnostic and therapeutic practice and to discuss possible future implications.

## 2. Pathogenesis of Adrenocortical Cancer

The adrenal cortex is divided into three zones: zona glomerulosa, zona fasciculata and zona reticularis where three main pathways of steroidogenesis occur. Adrenocortical carcinoma is a rare malignancy originating from the cortex of the adrenal gland with a poor prognosis due to its aggressive nature and unresponsiveness to conventional chemotherapeutic strategies. Although most ACC cases are sporadic and without a known cause, a minority of cases occur within other syndromes. The most common of these are Li-Fraumeni syndrome (*TP53* gene germline and somatic mutation), Lynch syndrome (*MSH2, MLH1, MSH6, PMS2, EPCAM* genes), multiple endocrine neoplasia type 1 (*MEN1* gene), Beckwith–Wiedemann syndrome (*11p151* gene, IGF-2 overexpression), familial adenomatous polyposis (*FAP* gene, β catenin somatic mutations), neurofibromatosis type 1 (*NF1* gene) and Carney complex (*PRKAR1A* gene) [5,7,9]. In spite of evident progress, molecular mechanisms of ACC tumorigenesis have not been yet fully understood [10]. Several molecular alterations and signaling pathways are thought to have a main role in tumor development. Monoclonality indicates that tumor progression is the end result of an intrinsic genetic tumor driver mutation [11]. Most common mutations implicated in sporadic ACC are insulin-like growth factor 2 (IGF2), β-catenin (CTNNB1 or ZNRF3) and TP53 mutations [12,13,14].

The main proposed oncogene in ACC tumorigenesis is insulin-like growth factor 2. The IGF-2 gene is located at 11p15 region that consists of a telomeric domain including the IGF-2 and H19 that might modulate IGF-2 expression and a centromeric domain including cyclin dependent kinase inhibitor (CDKNIC) involved in the G1/S phase of the cell cycle [11]. IGF-2 gene encodes IGF-2 protein and it is expressed by both fetal and adult adrenal glands and as a part of complex signaling system which plays an important role in normal growth and development, cell survival and proliferation as well as in malignant alteration [15]. IGF-2 overexpression was proven in more than 85% of ACCs although it is low or absent at the beginning of clonal proliferation [16]. Different studies have shown that IGF2 mRNA expression was 10–20-fold higher and IGF2 protein expression 8–80-fold greater in ACC compared to normal adrenal glands or adrenocortical adenomas (ACA), speculating that different IGF2 concentrations could be responsible for different biological behaviors of ACC [17,18,19,20,21,22]. IGF2 activates tyrosine kinase receptors that in turn lead to mitogen-activated protein kinase (MAPK) and phosphatidylinositol 3-kinase (PI3K)/Akt pathway activation. Activated Akt is then able to trigger the subsequent activation of the mammalian target of rapamycin (mTOR) pathway [22]. These pathways are involved in proliferation, survival, and metastasis of cancer cells [22].

Another signaling pathway included in ACC tumorigenesis is the canonical Wnt/β-catenin pathway where β-catenin protein plays a central role. The Wnt signaling pathway is normally activated during embryonic development where β-catenin stimulates and maintains proliferation of adrenal cortical cells, but it is also required for cell renewal in the adult adrenal cortex [23]. It has a structural role in cell–cell adhesion, and it is a transcription cofactor with T-cell factor/lymphoid enhancer factor mediating transcriptional activation of target genes of the Wnt signaling pathway [23]. Constitutive activation of Wnt/β-catenin is involved in many tumor types and, in experimental studies, it has been shown to act as an adrenal oncogene [24]. The Wnt/beta-catenin pathway in ACC can be activated by *CTNNB1* mutations and by *ZNRF3* (zinc and ring finger protein 3) inactivation [24]. According to the data from the literature almost 50% of ACCs show increased cytoplasmic or nuclear β-catenin accumulation [25].

Tumor protein 53 (TP53, p53) is a protein product of tumor suppressor gene located on chromosome 17 (17p13.1). P53 plays a role in regulation of the cell cycle, apoptosis, genomic stability and activation of DNA repair proteins. It is the most frequently altered gene in sporadic cancers, with greater than 50% of human tumors harboring somatic mutation [11,26]. According to genomic analyses, germline mutations in TP53 were observed in 50–80% of children with sporadic ACC, while somatic TP53 mutation was observed in 20% to 30% of sporadic ACC patients where it correlates with poor outcome [27]. In immunohistochemical studies diffuse p53 staining correlates positively with increased Ki-67 expression [28].

## 3. Hormonal Work-Up

Adrenocortical carcinomas may be functional—producing several types of steroid hormones with a pattern of disorganized steroidogenesis probably due to decreased expression of enzymes in undifferentiated tumors, or nonfunctional—presented as an adrenal mass without hormone excess [7,8]. Biochemically or clinically apparent adrenocortical hormone production is evident in up to 45% to 70% cases and by using more sensitive analytic methods, steroid hormones excess can be diagnosed in up to 95% of ACC cases, even without evident clinical presentation [14]. Hypercortisolism is the most common (in 50%–80% cases) and it is characterized with symptoms of Cushing syndrome, i.e., facial plethora, hypertension, glucose intolerance or diabetes mellitus, muscle weakness/atrophy, central obesity, acne, hirsutism and osteoporosis [1,7,29,30]. Hypokalemia and hypertension might be present as a consequence of very high cortisol levels—mediated mineralocorticoid receptor activation exceeding the inactivating capacity of corticosteroid 11β-dehydrogenase isoenzyme 2 [31]. Meta-analysis has shown that cortisol-secreting ACC are associated with a worse overall survival, although the mechanism is still unclear [32].

On the contrary, although autonomous cortisol production without clinically overt Cushing’s syndrome is a common finding in patients with adrenal incidentalomas, the rate of subclinical Cushing’s syndrome in ACC has not been widely studied. Subclinical hypercortisolism is defined as alteration of the hypothalamus–pituitary–adrenal axis in the absence of clinical, signs or symptoms related to cortisol secretion. It can be named as subclinical (or pre-clinical) Cushing’s syndrome, subclinical autonomous glucocorticoid hypersecretion or subclinical hypercortisolism. Beside the Italian Society of Endocrinology study that has revealed four cases of subclinical Cushing’s syndrome out of 47 ACC cases (8.5%), in the literature mostly only individual case presentations can be found [33,34,35,36].

Beside cortisol, hyperproduction of other hormones could be observed, such as androgens in 20% to 60% of cases, estrogen in 6% to 10% of cases, aldosterone in 2% of cases, simultaneously androgen and cortisol in 50% of patients or rarely all of them [7]. Clinical presentation of excessive adrenal androgens production in females includes rapid-onset male pattern baldness, hirsutism, menstrual abnormalities, early puberty, post-menopausal bleeding and virilization which was reported in 24%–55.1% of all ACC [1,7,37]. On the contrary, in males, androgen secreting tumor is often misdiagnosed because of isolated hyperandrogenism, but if peripheral conversion of androgens to estrogens or their co-expression occur, signs include gynecomastia, diminished libido and testicular atrophy [1]. Aldosterone-producing ACC is scarce, resembling primary hyperaldosteronism with an increase in plasma aldosterone on average by 14-fold and for a half-suppressed plasma renin activity with consequential hypokalemia and hypertension.

Other uncommon ACC associated abnormalities include hyperreninemic hyperaldosteronism, erythropoietin-associated polycythemia, chemokine release induced leukocytosis and IGF-2–mediated hypoglycemia named Anderson’s syndrome [1,38,39,40]. Non-functioning adrenocortical tumors are often associated with a high prevalence of insulin resistance and compensatory hyperinsulinemia and metabolic syndrome that may play a role in adrenal tumor growth through the activation of insulin and IGF-1 receptors [41]. Else and Nakamura with their co-authors have emphasized that, regardless of size, in the evaluation of adrenal tumors, androgen or estrogen production, development of rapidly progressive Cushing syndrome and concurrent hypersecretion of multiple hormones or precursors should always raise the suspicion of a malignant etiology [1,7].

In biochemical diagnostic procedure, the first step is the measurement of steroid hormones which is initially guided with clinical presentation. According to the ESMO-EURACAN (European Society for Medical Oncology—the European Reference Network for rare adult solid cancers) Clinical Practice Guidelines from 2020 in cases of suspected ACC, an extensive steroid hormone work-up is recommended assessing gluco-, mineralo-, sex- and precursor-steroids [42]. For all adrenal masses, diagnosis of pheochromocytoma should be excluded by measuring plasma-free or urinary-fractionated metanephrines to avoid intraoperative complications [42,43] (Table 1).

Cortisol hyperproduction causes suppressed ACTH (<10 pg/mL) and increased morning cortisol levels. The diagnosis of hypercortisolism is usually established by a 1-mg dexamethasone suppression test (1-mg DST), midnight salivary cortisol, or elevated 24-h urine free cortisol [44]. Autonomic cortisol secretion is confirmed for cortisol levels above 5 µg/dL after 1-mg DST, whereas a value < 1.8 µg/dL is indicative of normal adrenal function [31]. A value >1.8 µg /dL measured after high-dose dexamethasone suppression test (8 mg overnight or 0.5 mg every 6 h for 2 days) performed for “grey zone” speaks in favor of the diagnosis of Cushing syndrome [45]. Patients with subclinical hypercortisolism may have normal urine cortisol values but an elevated late-night salivary cortisol concentration.

Dehydroepiandrosterone sulfate and total or bioavailable testosterone measurements are recommended in every patient [1]. Plasma renin activity, serum aldosterone levels (a cut-of value > 10 to 15 ng/dL is recommended in the literature) and serum aldosterone to plasma renin ratio (ARR) are useful diagnostic tools for aldosterone producing ACCs [45]. ARR > 20–30 is indicative of hyperaldosteronism [45]. Because of increased production of deoxycorticosterone, pseudo-aldosteronism also can be observed [31]. However, it is important to keep in mind that regardless of ACC size, due to insufficient or aberrant steroid hormone synthesis, symptoms of hormonal hyper production or blood hormone levels can be absent or just moderately elevated [1].

## 4. Steroid Metabolome Profiling

Preoperative differentiation between adrenocortical adenomas and carcinomas, due to their opposed biological behavior, in clinical practice is indispensable, but limited to imaging and biochemical analysis [46]. Although ACC can be hormonally active, precursors and metabolites, rather than end products of steroid biosynthesis, are often accumulated [47,48,49]. Namely, these precursors are produced by dedifferentiated and immature malignant cells and represent intermediate steps of the major pathways of adrenocortical steroidogenesis [48,49]. Latterly, plasma and urine steroid metabolome profiling, including measurements of androgen, glucocorticoid and mineralocorticoid precursors and metabolites, as a specific steroid fingerprint, with respect of individual heterogeneity, has been imposed as a marker of distinguishing malignancy in adrenal masses [50,51]. Although most of these metabolites are not measured routinely, they can be detected by novel techniques.

The traditional approach with a separate immunoassay was complicated, time consuming and costly using individual aliquots of serum to analyze each substance separately [52]. Further progress was achieved by using gas chromatography/mass spectrometry or liquid chromatography–tandem mass spectrometry (LC–MS/MS) analysis. McDonald et al. in their review have pointed out that the appearance of commercial high-performance liquid chromatography instruments linked to tandem mass spectrometers, as a faster and more available method, offers the potential for medium-to high-throughput profiling using small amounts of sample in diagnosing ACC accentuating evaluation of multiple hormones and precursors simultaneously, while for many years utilized gas chromatography/mass spectrometry profiling was not widely available and was characterized by slow throughput [46,52,53]. A similar conclusion was made by Rossi et al. in the recent published review emphasizing that LC-MS/MS steroid profiling could be the most informative test in the initial diagnostic approach of adrenal tumors with high specificity and sensibility based on a single analysis and provides backing for the further costly genetics tests [54].

According to the literature, elevated serum levels of 11-deoxycortisol, and/or its glucuronated metabolite tetrahydro-11-deoxycortisol, pregnenolone sulfate, 17α-hydroxypregnenolone sulfate and androstenedione are highly discriminative for ACC [46,51,53,55]. While 11-deoxycorticosterone, progesterone and estradiol were significantly different in both males and females, aldosterone was lower in males, but 17 OH-progesterone, dehydroepiandrosterone (DHEA), dehydroepiandrosterone sulfate (DHEAS) and testosterone were higher in females [46]. Respecting serum steroid measurements variability because of the diurnal variation, a diagnostic preference was given to urinary steroid metabolomics examinations [56].

**Figure 1:**
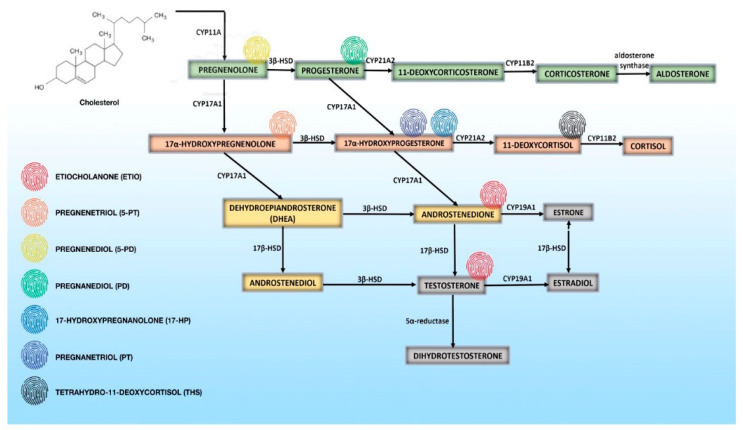
Overview of normal steroidogenesis and urinary steroid metabolites i.e., “malignant steroid fingerprint” indicative of adrenocortical carcinoma [56,68,69,70].

Numerous studies have been published aiming to distinguish the malignant potential of adrenal tumors according to analysis of adrenal steroidogenesis products [46,47,48,49,50,51,53,55,57,58,59,60,61,62,63,64,65,66]. The cornerstone of more recent steroid profiling studies was the one of Arlt et al. where the authors have concluded that metabolites of 11-deoxycortisol and DHEA are the most useful for this purpose [47]. This study defined the 11-deoxycortisol metabolite tetrahydro-11-deoxycortisol (THS) as the most discriminative marker [47]. Kerkhofs et al. later published results of 15 steroid metabolites that had a sensitivity > 90% for detecting ACC, of which 7 had a sensitivity of 100% [59]. Those are tetrahydro-11-deoxycortisol (THS), pregnanediol (P2), pregnanetriol (P3), etiocholanolone (E), androsterone, tetrahydrocortisol, and tetrahydrocortisone [59]. It is important to emphasize that there were no significant differences in individual metabolite excretion between the groups with functioning ACA and non-functioning ACA [59]. In benign and malignant tumor differentiation, highly suggestive of ACC are excretion of unusual steroid metabolites which are products of aberrant steroidogenesis, but also the reappearance of neonatal steroid metabolites after regression of the adrenal fetal zone, such as 16 α–hydroxypregnenolone [67]. The most recent multicenter study included 2017 participants from 11 countries and among them 98 patients had confirmed ACC diagnosis [68]. Seven steroid metabolites comprised in the “malignant steroid fingerprint” indicative of ACC identified by machine learning in a previous study were confirmed [69]. These are as follows: etiocholanolone, pregnenetriol, pregnenediol, pregnanediol, 17-hydroxypregnanolone, pregnanetriol and tetrahydro-11-deoxycortisol [68] (Figure 1).

Suzuki et al. have correlated steroid profiling results with pathological factors [64]. Their results have shown positive correlation between glucocorticoid precursor 11-deoxycortisol with predictive prognostic factors of European Network for the Study of Adrenal Tumors (ENSAT) classification, while testosterone levels positively correlated to the Ki67-index [64]. Sun et al. in 2019. have proposed novel tool tissue-based chemical phenotyping MALDI mass spectrometry imaging (MALDI-MSI) that offers new insights in the pathophysiology of ACC [71]. They have shown that estradiol sulfate, estrone 3-sulfate and expression of sulfotransferase SULT2A1 were significantly associated with prognosis of ACC [71]. Authors have discovered the presence of estradiol-17β 3,17-disulfate (E2S2) in a subset of tumors with particularly poor overall survival. However, not only steroid precursors, but other (un)known metabolites might be useful in distinguishing ACC from ACA. In the experimental study of Patel et al. urinary creatine riboside was elevated 2.1-fold, and L-tryptophan, Nε,Nε,Nε-trimethyl-L-lysine, and 3-methylhistidine were lower 0.33-fold, 0.56-fold, and 0.33-fold, respectively, in patients with ACC (all *p* < 0.05) [65]. Based on that discussed above, steroid metabolome profiling is definitely a promising diagnostic tool in preoperative assessment of malignant potential of adrenal tumors, but further studies are needed as well as even wider integration of the method in more specialized laboratories.

## 5. Pathological Approach to Adrenocortical Carcinoma

Adrenocortical tumors are a unique group of tumors whose differentiation between adenomas and carcinomas is a great challenge even for pathologists since no single pathohistological marker indicates malignancy [72]. Pathological assessment, crucial for the diagnosis of ACC after surgical resection relies on morphological features, margin identification and immunohistochemical staining [73]. Biopsy of a specimen of adrenal tumors is usually contraindicated due to possible complications and the fact of it not being completely informative [42]. Relative indication remains to exclude/prove secondary etiology of non-functional adrenal tumor in patients with positive anamnesis of extra-adrenal neoplasm [42]. Weight and size of the resected tumor should be the first to raise the suspicion for malignancy [72]. In the literature, different cut off values can be found to determine it: >95, >50, >100 g, but also in some cases tumors <50 g had malignant potential [72,73,74,75]. Most morphological studies confirm the size of the malignant lesion to be greater than 50–65 mm, ranging from 20 to 196 mm [72,73,74,76]. It is important to keep in mind that tumor size might be underestimated by radiological investigation and not correlate with the real size of tumor lesion [76,77]. Except that above mentioned, further examination should include evaluation of capsule integrity and the presence of hemorrhage, necrosis and invasion [76].

Beside the classical form, adrenocortical carcinoma can have other rare histological variants like oncocytic, myxoid and sarcomatoid [78]. ACC arise from the different zones of the adrenal cortex and they most often have the cellular morphology characteristic of different adrenocortical cells [73]. According to the latest guidelines, immunohistochemical panel staining should be done, including steroidogenesis factor 1 (SF1), adrenocortical-specific marker or alternatively inhibin-alpha, calretinin and melan-A for identification of adrenocortical tumors, chromogranin A for identification of pheochromocytoma and paraganglioma as well as synaptophysin for both [42]. Adrenocortical cells express SF-1, a transcriptional factor, during fetal and adult life, mostly in the zona glomerulosa and fasciculate [79]. Experimental studies have confirmed that its high expression positively correlates with high mitotic count, high Ki-67 index, and high European Network for the Study of Adrenal Tumors (ENSAT) stage and negatively with loss of functionality, presence of oncocytic features and decreased survival [79]. Therefore, steroidogenic factor 1 can be used as diagnostic and prognostic marker in adrenocortical carcinoma [79,80].

Ki-67 is also routinely measured and, although nonspecific for ACC, it has a prognostic role. Ki-67 is a protein expressed in all cell cycle phases except G0 and represents a cell proliferation index. Ki-67 labeling index of more than 5% confirms the diagnosis of ACC [7,76]. Ki67 index >10% correlates with higher risk of recurrence in ACCs and it is associated with worse overall survival in patients with advanced disease or rapid disease recurrence [76,81]. Although practical utility of Ki-67 staining was indisputable and confirmed in many studies, one should keep in mind that it is hard to set a diagnostic threshold because of possible interobserver variations [82]. According to some authors, a combination of insulin-like growth factor 2 (IGF2) and Ki67 index might be useful for differentiating malignant etiology of adrenal masses [83,84]. Beside abovementioned markers, steroidogenic enzymes, p53, cyclin E and β-catenin expression might be also histologically analyzed [7]. Several novel markers and some other roles of already known biomarkers were investigated in experimental studies using immunohistochemistry (± other methods) on a different number of patients with benign and malign adrenal tumors. The aim of analyses was to elucidate their utility in the diagnostic approach of discriminating malignant lesions, to investigate possible pathophysiological role and, finally, to analyze their prognostic and targeted therapy efficiency (Table 2). Further studies on larger cohorts are needed for their implementation in routine praxis.

ACCs can be graded into low- and high-grade based on their mitotic rates (≤20 mitoses per 50 high-power fields (HPF) or >20 mitoses per 50 HPF [1]. In clinical practice, several scoring systems have been developed to help distinguish malignant from benign adrenal tumors. The most widely used diagnostic tool is the Weiss score. The Weiss score includes nine histopathological parameters, related to tumor and cellular structure as well as invasion. A score of ≥3 suggests malignancy [76]. For an oncocytic variant of ACC Lin–Weiss–Bisceglia (LWB) scoring is proposed and Wieneke criteria are more reliable than Weiss scoring for the pediatric population [121,122,123]. They are all systematically shown in the Table A1 in Appendix A.

Another simplified diagnostic algorithm termed the Reticulin algorithm was proposed several years ago, with a sensitivity and specificity of 100% for ACC [83]. It includes evaluation of disruption of the reticular network (highlighted by histochemical staining) and at least one of following parameters: mitotic rate >5/50 high-power fields, necrosis and vascular invasion [83]. In 2015, the Helsinki score was developed for more precisely predicting occurrence of metastases in adrenocortical carcinoma [124]. According to Duregon et al., who performed analysis on 225 ACC patients, it presents the most useful tool with an impact on prognosis, outperforming other prognostic parameters such as clinical stage, mitotic index and Ki-67 proliferation index, also applicable in all histological variants of disease [78]. The Helsinki score accounts for morphological (mitoses and necrosis) and immunohistochemistry parameters (the absolute value of the Ki-67 proliferation index), meaning 3× mitotic rate greater than 5/50 high-power fields + 5× presence of necrosis + proliferation index in the most proliferative area of the tumor [78]. With a cut off value of 8.5, this scoring has 100% sensitivity and 99.4% specificity for diagnosing metastatic ACC [83,124]. In summary, the Helsinki and Weiss score are predictors of poor prognosis, while the Helsinki score and Ki-67 index are the best predictors of disease-related death [78]. It is important to mention that in different studies, some other cut off values of the abovementioned scores were proven, i.e., <13 and ≥19 for the Helsinki score [78]. Further studies are needed to elucidate this area and its reproducibility.

## 6. Circulating Tumor Biomarkers

Tumor marker, produced by adrenocortical cancer cells providing information about tumor presence, malignancy potential, aggressiveness, therapeutic response, probability of tumor recurrence or counting diseases free survival with a reliable predictive value, unfortunately, does not exist. This hypothetical marker of ACC should be effective, easily analyzed, with high sensitivity and specificity, but cheap and widely available. Worldwide, scientists are engaged in great efforts to discover potential diagnostic or prognostic markers although serum markers are still lacking. The overwhelming outcome is adrenocortical cancer heterogeneity, rarity and generally short survival.

The neutrophil-to-lymphocyte ratio (NLR) is an accessible and simple tool that has been examined as a biomarker for some solid malignant tumors for the last several years. Preoperative NLR ≥5 in ACC has been evaluated as a diagnostic and prognostic biomarker. Results have shown significantly higher values in comparison with the non-malignant group, correlating with poorer overall survival [125]. Similar analysis was performed among patients who underwent resection for recurrent ACC. The preoperative lymphocyte-to-monocyte ratio (LMR) was calculated. LMR >4 and time-to-recurrence >12 months correlated with longer disease-specific survival which could influence the decision on the therapeutic approach [126]. The most recent study has emphasized the utility of hemocytometer parameters in differentiating adrenal adenomas from carcinomas [127]. Significantly higher values of following parameters were noticed in ACC: neutrophil count, neutrophil/lymphocyte ratio, platelet/lymphocyte ratio, and red blood cell distribution width, while lymphocyte count, plateletcrit, hemoglobin and hematocrit were higher in the ACA group [127].

Results of the study of hormonal and metabolic disorder connections in ACC patients have revealed higher tumor necrosis factor alpha (TNF-α), interleukin 6 (IL6) and monocyte chemoattractant protein 1 (MCP1) levels [128]. It is possible that higher pro-inflammatory cytokine concentrations comprise an additional cardiovascular, metabolic and perhaps malignancy risk in these patients [128]. High IL-6 high level might stimulate the secretion of glucocorticoids and the serum level of matrix metallopeptidase 9, which is related to the cancer pathology including invasion, metastasis and angiogenesis [129]. Matrix metalloproteinase (MMP), are calcium-dependent zinc-containing endopeptidase playing an important role in tissue remodeling associated with various physiological or pathological processes including metastasis. Serum MMP-1, MMP-8 and MMP-9 levels were evaluated in patients with adrenal tumors prior to and after surgery. High levels of MMP-8 and MMP-9 levels were found in patients with adrenocortical cancer, but were not indicative in differing malignancy. However, MMP-8 and MMP-9 levels were not increased in patients with inoperable adrenocortical cancers while MMP-1 level was not increased in patients with either benign or malignant adrenal tumors. After surgery, MMP-8 and MMP-9 levels decreased significantly in patients with adrenocortical carcinoma, whereas the decrease in these MMPs in patients with benign tumors was not significant [130].

Ghrelin, often called a “hunger hormone” also has a role in cancer progression and studies have shown ghrelin and IGF2 overexpression in adrenal carcinoma with the possible role of a proliferative factor [10]. Levels of serum retinoic acid receptor responder protein 2 (RARRES2), known as chemoattractant and adipokine and, according to recent findings, a tumor suppressor by promoting β-catenin phosphorylation/degradation and inhibiting p38 phosphorylation in adrenocortical carcinoma, is elevated in patients with ACC. Paradoxically the RARRES2 gene has been found to be transcriptionally downregulated, as well as its tissue expression in ACC [107,131]. Inhibin A is a heterodimeric glycoprotein hormone expressed in the gonads and adrenal cortex members of the transforming growth factor-beta family of growth and differentiation factors. Since the adrenal cortex produces the inhibin α-subunit, the role of serum inhibin pro-αC as a tumor marker for ACC was analyzed [132]. ACC patients had higher serum levels than controls with sensitivity of 59% and specificity of 84% for differentiation from those with adenomas [132]. Another analyzed marker was serum glucocorticoid kinase 1 (SGK1), a glucocorticoid-responsive kinase involved in multiple cellular functions [133]. Low SGK1 expression was connected to ACTH-independent cortisol secretion in adrenocortical tumors and might be considered as a new prognostic factor in adrenocortical carcinoma [133]. In a report of a patient case with ACC, a high level of serum neuron-specific enolase (NSE) was noticed before operation and was considered as useful marker for monitoring tumor status during management [134]. Generally, NSE is a highly specific marker for neurons and peripheral neuroendocrine cells and it is an index of neural maturation. In this report, immunohistochemical analysis has shown positivity for NSE and overexpression of p53 [134]. Finally, a novel germ line variant of the 177 mutant (Pro to Arg; P177R) of p53 by genomic sequencing was then identified [134].

## 7. Genetic Analysis

Over several decades, a great effort has been invested in comprehensive and integrated genome investigation of adrenocortical carcinoma, making a step forward towards personalization of cancer medicine. ACC is characterized with genetic diversity and heterogeneity. The aim of molecular studies is to identify additional oncogenic alterations, providing a fundamental basis for translational researches and development of novel therapies. Genomic studies have managed to distinguish ACC subgroups as well as malignant biological behavior, analyzing specific molecular alterations, with regard to DNA level somatic mutations, chromosome alterations, DNA methylation transcriptomes, the whole exome sequencing and miRNome [135]. ACCs arise from mutation-induced monoclonal cell populations [136,137]. High prevalence of aneuploidy in ACC suggesting chromosomal instability has also been noted [1]. Genomic abnormalities at chromosomes 5, 12, and 17 are predicted to be cornerstone in ACC tumorigenesis [1]. DNA hypermethylation of promoters correlates with poor survival and can distinguish carcinomas from adenomas with a sensitivity of 96% and specificity of 100%, highlighting a possible role of methylation in the 11p15 locus containing IGF2 and H19 as a valuable biomarker [14,138,139,140]. While somatic mutation can differ, prognostic DNA methylation and chromosome alteration profiles seem rather stable and might be more powerful for the prognostic evaluation [135]. It is an independent prognostic marker, compatible with ENSAT stage and Ki67 proliferation [138].

### 7.1. Genome Sequencing

Next generation DNA sequencing (NGS) has brought about revolution in genomic research. In the literature, numerous reports can be found proposing comprehensive and novel molecular pathophysiological, diagnostic, therapeutic and prognostic genes associated with ACC [4,50,113,141,142,143,144,145,146,147,148,149,150,151,152,153,154,155,156,157,158,159,160]. Zheng et al. have summarized proposed genes as potential drivers involved in sporadic adrenocortical tumorigenesis, including insulin-like growth factor 2, β-catenin (CTNNB1), TP53, ZNRF3 and TERT, as well as novel nominated drivers such as PRKAR1A, RPL22, TERF2, CCNE1 and NF1 [161]. IL13RA2, HTR2B, CCNB2, RARRES2 and SLC16A9 genes are not just dysregulated in ACC, but also have excellent diagnostic accuracy for distinguishing benign from malignant adrenocortical tumors [162].

Genomic sequencing of 29 ACC samples was performed by Ross et al. to identify potential targets of therapy and analyze genomic alterations (GAs) for relapsed and metastatic ACC [163]. At least one GA was found in 76% ACC and the most frequent were in TP53, NF1, CDKN2A, MEN1, CTNNB1 and ATM [163]. Authors have emphasized that in 59% of ACC at least one GA was associated with an available therapeutic option [163]. Alshabi et al. have identified 884 differentially-expressed genes in ACC, from which 441 are up-regulated and 443 down-regulated [164]. From these, hub genes, i.e., genes with the highest correlation in candidate modules, were YWHAZ, FN1, GRK5, VCAM1, GATA6, TXNIP, HSPA1A, and F11R [164]. YWHAZ, STAT1, ICAM1, SH3BP5, CD83, FN1, TK1, HIST1H1C, CABLES1 and MCM3 genes were associated with poor overall survival, while STAT1, ICAM1, CD83, FN1, TK1, HIST1H1C and MCM3 were highly expressed in stage 4 of ACC [164]. The important conclusion was made by Fojo et al. whose results have shown that genomic aberrations of advanced and metastatic tumors were similar to those from newly diagnosed patients providing novel directions in this research [165]. Interestingly, dysregulation of iron metabolism-related genes has been characterized as a promising prognostic biomarker in cancers, including ACC [166]. Namely, reduced expression levels of ferroportin1 (FPN1) and ceruloplasmin (CP) were found in ACC patients and significantly correlated with poor survival. Moreover, expression levels of FPN1 negatively correlated with the pathological stages of ACC [166].

Another meta-analysis of pan-genomic studies was performed in 368 ACC patients, analyzing targeted gene expression (BUB1B and PINK1), methylation (PAX5, GSTP1, PYCARD, and PAX6), and next-generation sequencing [167]. The main aim was to measure the prognostic value of each model. Results have shown that molecular class was an independent prognostic factor of recurrence in stage I to III ACC after complete surgery and, interestingly, with limited benefit in stage IV [167]. Li et al. have correlated adverse prognostic genes with tumor microenvironment (TME) [168]. Authors have analyzed 1649 differentially expressed genes (DEGs) and 1521 DEGs based on immune and stromal scores and have found positive correlation among them [168]. Expression of differentially expressed immune-related genes (IRG) in ACC was analyzed using several genome databases. To predict immune cell infiltration, an immune score was calculated using ESTIMATE (Estimation of Stromal and Immune cells in Malignant Tumor tissues with Expression data). A high immune score predicted a good prognosis and an early clinical stage in ACC [129]. Results have shown that the five most significant signaling pathways for activation of the differentially expressed IRGs were the PI3K–Akt, JAK–STAT, chemokine signaling pathways, and the Ras and MAPK signaling pathway [129]. Analysis has identified 30 IRGs associated with survival [129]. Among all of them, centromere protein A (CENPA), E2F transcription factor 1 (E2F1) and forkhead box M1 (FOMX1) have shown upregulated expression that was involved in ACC progression and were predictors of worse outcome. In contrast, downregulation of transcription factor 21 (TCF21) expression resulted in the accumulation of secreted glucocorticoids and accelerated proliferation of ACC cells [129].

The first study of weighted gene co-expression network analysis (WGCNA) algorithm analysis to construct a gene co-expression ACC network associated with tumor grade and poor prognosis was published in 2018 [169]. Results have accentuated twelve hub genes (ANLN, ASPM, CDCA5, CENPF, FOXM1, KIAA0101, MELK, NDC80, PRC1, RACGAP1, SPAG5, TPX2) which have good distinctive power for malignancy and correlate with unfavorable prognosis and tumor stages [169]. With bioinformatics analysis highly associated with the cell cycle, organelle fission, chromosome segregation, cell division and spindle stability, 71 genes were reported [170]. Beside the abovementioned, these are BIRC5, CDK1, EZH2, MAD2L1, NCAPG, PBK, RRM2 and TOP2A [170]. The nuclear division cycle 80, cyclin B2 and topoisomerase 2-α are possibly included in tumor development, predict overall survival and recurrence-free survival in patients with ACC [170]. Furthermore, occurrence of massive DNA loss followed by whole genome doubling (WGD) can occur and it is associated with aggressive clinical course, suggesting WGD is a mark of disease progression [161]. The most recent next generation sequencing analysis aimed to correlate genome alterations with additional therapy options in refractory ACC [171]. A panel of 592-gene DNA-based profiling was performed from 94 (primary versus metastatic disease) cancers [171]. The most frequently mutated genes were TP53 (36%) and CTNNB1 (19%) while low prevalence mutations were noted in 37 genes including DNA damage repair genes [171]. Potential targets to approved drugs were seen in only 16% [171]. Another step to targeted treatment was identification of oncogenic driver gene set (ZFPM1, LRIG1, CRIPAK, ZNF517, GARS and DGKZ), involved in tumor suppression and cellular proliferation [172].

### 7.2. MicroRNA

MicroRNA (miRNA) is a short single stranded non-coding RNA molecule involved in the epigenetic regulation of cellular processes [173,174,175]. MicroRNAs regulate gene expression by inhibiting mRNA translation or degrading mRNA transcripts [176]. One third of coding genes are regulated by miRNAs so they are implicated in practically every biological process [6,177]. Several studies have shown that various circulating or tissue microRNAs can differentiate ACC from benign tumors [177,178,179,180]. Not only as a biomarker of ACC, microRNAs also provide a potential therapeutic target. One of the first studies in seven proven ACC using miRNA profiling was published in 2009, profiling 368 miRNAs [181]. The authors have revealed that by setting the cut-off value of ΔCT miR-511–ΔCT miR-503 at 1.4, malignant tumor can be accurately distinguished from benign adrenal mass with 100% sensitivity and 80% specificity [181].

MiR-483-5p is one of the most investigated miRNAs in ACCs, both as a diagnostic and prognostic biomarker and has been proven as the best single-gene malignancy marker [182]. Since miR-483-5p is located at 11p15.5 within the second intron of IGF2, the high expression of miR-483-5p observed in ACC may be an indirect consequence of IGF2 over-expression [177,183]. Expression levels of six microRNAs including miR-503-5p, miR-483-3p, miR-450a-5p, miR-210, miR-483-5p and miR-421 can predict malignant potential with at least 95% accuracy [182]. High circulating levels of miR-483-5p or low circulating levels of miR-195 are associated with both shorter recurrence-free survival and shorter overall survival in ACC [184]. Serum miR-483-5p levels measured 3 months after surgical procedure were, for example, higher in those with recurrence disease or lethal outcome within three years than in those without [185]. Receiver operating characteristic analysis showed that a value of 752,898 copies/mL was a cut off value to prognosticate recurrence with 61.5% sensitivity and 100% specificity [185]. Beside circulating miR-483-5p, its urinary counterpart has been evaluated in patients with adrenal tumors [186]. However, no significant difference was detected between ACC and ACA urinary samples, although already specific plasmatic overexpression of hsa-miR-483-5p was proven [186].

### 7.3. Novel Genomic Approaches

Another perspective area of ACC investigation is the analysis of circulating tumor cells (CTCs). CTCs are neoplastic cells originating from either the primary tumor or metastases and they are isolated from peripheral patients’ blood [173]. CTCs’ role has been evaluated in several malignancies as a prognostic biomarker [173]. Studies have confirmed that CTCs are present in the blood stream of patients with ACC, but not in those with adenomas [187]. Significant decrease in the number of CTCs has been reported after operative treatment compared to levels prior the procedure, indicating drop of the CTCs number after the mass removal [187]. The results of the recently published study have shown that CTCs, obtained from liquid blood biopsy, were found in 68% of pre-surgery and in 38% of post-surgery blood samples [188]. Stratifying patients in high and low pre-surgery CTC number groups (75th percentile CTC value as the cut-off), CTCs significantly predicted overall survival [188].

Study of cell-free DNA (cfDNA), various forms of DNA freely circulating in the bloodstream, including cell-free DNA (cfDNA) and circulating tumor DNA (ctDNA) has generated interest in ACC research. Their role of a tumor dynamics predictor has been investigated in some types of cancers as a consequence of inflammation and cell death. In pilot studies of several ACC patients, a blood sample was taken 1–2 weeks prior and after surgery and cell-free circulating DNA (cfDNA) was isolated [189]. Tumor-specific mutations were found in the cfDNA of one of the three patients who had metastatic ACC at diagnosis. The preoperative cfDNA showed the same mutations as by NGS, both pre- and postoperative, but in the latter with lower frequencies [185]. Furthermore, ctDNA, i.e., fragments of DNA released directly by tumor cells into the blood stream is discriminated from other non-tumoral cf-DNA by the detection of somatic mutations, specific of cancer cells, tumor type and stage [190]. If detected, ctDNA followed tumor dynamics in a small cohort of patients with ACC, but further studies are needed on a larger population [190].

Long non-coding RNA (LncRNA) are transcripts of RNA of more than 200 nucleotides, with no protein coding potential [191]. LncRNAs have important functional roles in epigenetic, transcriptional or post-transcriptional regulation [192]. Their role has been evaluated in several autoimmune diseases and different cancer types. In adrenocortical cancers, long noncoding RNA expression profile can distinguish samples from adrenocortical carcinomas and control groups. Actually, a total of 874 long noncoding RNAs were differentially expressed between adrenocortical carcinoma and normal adrenal cortex and can correlate to unfavorable outcome [193]. Heterogeneous nuclear ribonucleoproteins (hnRNPs) comprise a family of RNA-binding proteins, involved not only in processing heterogeneous nuclear RNAs (hnRNAs) into mature mRNAs, but also acting as trans-factors in regulating gene expression [194]. Their implication in various aspects of tumorigenesis has been investigated. Recent discoveries have shown that most hnRNPs were associated with worse survival in ACC [195].

Finally, Xie et al. have developed Online consensus Survival analysis of ACC (OSacc), an easy-to-use, freely available interactive online tool of survival analysis based on seven independent transcriptomic profiles with long-term clinical follow-up information of 259 ACC patients (http://bioinfo.henu.edu.cn/ACC/ACCList.jsp (accessed on 10 February 2021)) [196]. In addition, Ye et al. have designed Advanced Expression Survival Analysis (AESA), a web tool using the rich gene expression data from The Cancer Genome Atlas (TCGA), supporting novel survival analysis approaches to the set of genes [197].

## 8. Treatment Options

Despite of its rarity, with global prevalence of 4–12 cases per million, adrenocortical carcinoma is the second, after anaplastic thyroid carcinoma, most lethal endocrine malignancy with 5-year overall survival being < 15% in advanced ACC [15,42,198]. The main obstacle is scarcity of effective and available treatment options [27]. Surgical complete resection is still the treatment mainstay with curative intensity but only in patients with localized tumor mass [199]. The most used drug for treatment of ACC is mitotane, an adrenolytic drug, a derivate from the insecticide dichlorodiphenyltrichloroethane, which was introduced in 1960 for this indication, both in an adjuvant setting and for advanced disease [151,200]. Mitotan targets enzyme sterol O-acyltransferase 1 (SOAT1) which is expressed in steroidogenic adrenal cells [201]. Although mitotane has been a cornerstone therapy for ACC, severe adverse effect and toxicity, as well as drug interactions, should be followed with critical reappraisal of treatment indication during the whole treatment process [202,203]. In rigorously selected patients, the response rate was up to 30% with a progression-free survival of 8.8 months and an overall survival of 29.6 months [200].

In metastatic disease, first line systemic therapy consists of etoposide, doxorubicin, cisplatin and mitotane (EDP-M); in the future potentially liposomal EDP-M regimens with improved tolerability could be created [199,204,205]. However, standard chemotherapy only has a 23% response rate [171]. Other treatment possibilities include radiofrequency ablation, transcatheter arterial chemoembolization, radiation therapy, cryoablation and microwave ablation for local tumor control as well as streptozotocin +/− mitotane, gemcitabine and capecitabine +/− mitotane, radionuclide treatment with [131I]-metomidate (I-MTO), trofosfamide, thalidomide, temozolomide, targeting the IGF-II/IGF receptor I pathway and tyrosine kinase inhibitors as systemic therapy [42,200,206,207,208].

Due to the disease’s rarity, heterogeneity, lack of registry bases and high cost of clinical investigations, data on the effectiveness of these interventions are limited [209]. Further prospective clinical trials are definitely needed because, despite the remarkable progresses made in understanding of the molecular signature in ACC, a major turning point in treatment success was not produced [210]. Altieri et al. have elaborated possible reasons for the disappointing results of new targeted therapies, such as insulin growth factor-1 (IGF-1), mammalian-target of rapamycin (m-TOR), vascular endothelial growth factor (VEGF) inhibitors and other options as a result of drug interactions with mitotane. Disease heterogeneity with exceptional responses in very few patients, absence of target mutation and resistance mechanisms to immunotherapy occurred [205,211,212]. ACC patients treated with Sunitinib, a tyrosine kinase inhibitor reached, in 14.3% of cases, stable disease after 12 weeks with a median overall survival of 5.4 months [200]. In this study, co-treatment with mitotane negatively impacted on the anti-tumoral effect and level of the drug due to mitotane-induced CYP3A4 [200]. However, another tyrosine kinase inhibitor, cabozantinib (CABO), which also targets tyrosine-protein kinase Met (c-MET), in monotherapy appears to be safe and effective in advanced stages of ACC [213].

Tumor cells can escape the immune response by using immune checkpoints, such as programmed death-1 (PD-1), programmed death ligand-1 (PD-L1) and cytotoxic T lymphocyte antigen-4 (CTLA-4) in the tumor microenvironment [129]. The concept of immune checkpoint inhibitor targeting was the framework of the development of the newest ACC therapies, especially in patients with advanced malignant disease. Since the first data of their effectiveness were controversial, appropriate pre-selection of patients might be the key (e.g., tumors that express PD1/PD-L1 or tumors with high mutational load [200]). Pembrolizumab is a humanized monoclonal antibody that targets the programmed cell death ligand 1 (PD-L1) pathway [214]. The results of a phase 2 trial have shown a non-progression rate at 27 weeks of 31%, objective response rate of 15% and clinical benefit rate of 54% [215]. Microsatellite-high and/or mismatch repair deficient (MSI-H/MMR-D) tumors, for which pembrolizumab is a standard therapy, are more common in ACC than has been recognized, according to Raj et al. [216]. The results of their study have reported a response rate to pembrolizumab of 23% and a disease control rate of 52%. The median progression-free survival was 2.1 months, and the median overall survival was 24.9 months with a good safety profile [214,216]. Efficiency of another checkpoint inhibitor, anti-PD-1 nivolumab, was investigated in ten patients with metastatic ACC [213]. Nivolumab demonstrated modest antitumor activity with median progression-free survival being 1.8 months [217].

### Experimental Studies

The cornerstone of diabetes mellitus therapy, metformin, has been proven to have anti-cancer effect in several solid tumors [218]. In experimental models of ACC, metformin was proven to reduce cell viability and proliferation in a dose- and time-dependent manner, trigger apoptosis and inhibit tumor growth [218]. It was also associated with a significant inhibition of the main signaling pathways already established in tumorigenesis of ACC [218]. These results were supported by a case presentation of patients remaining on maintenance therapy with metformin and melatonin whilst being free of disease for 7 years post diagnosis [219]. To come to a generally accepted conclusion, further investigations on a large cohort of ACC patients are urgent. Furthermore, mitotane use can cause hypercholesterolemia in patients with adrenocortical carcinoma and it is possible that cholesterol increases intratumor activity [220]. Simvastatin addition can reduce tumor volume and weight, prevent estradiol production and inhibit mitochondrial respiratory chain-inducing apoptosis in ACC cells [220].

In experimental studies on cell lines, C-terminal Hsp90 (heat shock protein 90) inhibitor KU758 has proven effectiveness as treatment for adrenocortical carcinoma cells upregulating long noncoding RNA expression for tumor suppression, including tumor suppressor GAS5, which is implicated in the β-catenin and mammalian target of rapamycin pathways [221]. Another study has proposed nicotinamide nucleotide transhydrogenase (NNT) which has a central role within mitochondrial antioxidant pathways, providing preclinical evidence of the therapeutic value of antioxidant targeting in ACC as well as illuminating the long-term adaptive response of cells to oxidative stress [222].

Rottlerin, a natural compound purified from *Mallotus Philippinensis*, is a specific protein kinase inhibitor [223]. Its effectiveness as an inhibitor of cellular proliferation, migration and invasion as well as a promotor of cell cycle arrest and apoptosis inducer of ACC cell lines has proposed rottlerin as a novel and potential chemotherapeutic agent in patients with ACC [223]. Another study has proven that nilotinib, a selective tyrosine kinase receptor inhibitor, as a cytotoxic drug that combined with ERK inhibitors deserves to be tested as a novel therapy options in ACC patients [224]. Palbociclib, the first cyclin-dependent kinase 4 and 6 (CDK4/6) inhibitor approved as a cancer therapy, causes a concentration- and time-dependent reduction in ACC cell viability, which was more pronounced in the cells in line with higher CDK4 expression [225]. Palbociclib in combination with insulin-like growth factor 1/insulin receptor inhibitor linsitinib shows an additive effect [225]. Hedgehog Receptor Patched is expressed in ACC and contributes to doxorubicin efflux and treatment resistance [226]. Utility of the anti-histaminergic drug astemizole, a new inhibitor of Patched drug efflux, was analyzed on ACC cell lines [226]. Astemizole at a low concentration sensitizes ACC cells to doxorubicin, magnifying its cytotoxic, proapoptotic and antiproliferative effects [226]. Withanolides, a group of naturally occurring polyoxygenated steroidal lactones built on an ergostane skeleton, are novel chemotherapeutic agents with potent targeted effects in medullary thyroid cancer and a number of solid malignancies with low toxicity in vivo [227,228]. In an experimental study on ACC cell lines, withanolides reduce ACC cell viability, induce cell cycle arrest and apoptosis as well as modulate expression of several key oncogenic pathway proteins [227].

The active vitamin D metabolite 1α,25-dihydroxyvitamin D3 (1α,25(OH)2D3) acts as an anti-proliferative agent in human cancer by inhibiting the Wnt/beta-catenin pathway through the vitamin D receptor (VDR). Mitotane and 1α,25(OH)2D3 have and additive effect on the inhibition of ACC cell growth and viability [229]. Nevanimibe HCl, a novel SOAT1 inhibitor, has been shown in experimental studies to decrease adrenal steroidogenesis at lower doses and to cause apoptosis of adrenocortical cells at higher doses [230]. However, it failed to show efficiency in ACC patients at obtained doses [230]. Results have shown that none of the patients experienced a complete or partial response, although several had stable disease [230].

## 9. Future Perspective

In spite of all the progress that has been evidenced, it seems that we are far away from reducing the ACC mortality rate and finding a unique ACC biomarker. Many questions still remain unanswered. Malignant potential among small adrenal incidentalomas < 4 cm; frequency of surveillance; ectopic extra-adrenal ACC presentation of an already, according to some authors, ultrarare disease; the possibility of advancement to adrenocortical carcinoma after decades in previously defined adenoma; risk factors of occurrence and many others doubts need to be kept in mind [231,232,233]. We might expect the increase in incidentaloma incidence due to technical improvement, frequency of use and availability of imaging methods, although distinguishing benign from malignant adrenal tumor with already-established diagnosis remains an enormous challenge. Precision medicine, with a personalized approach to every individual, is the only viable option in the successful fight with adrenocortical carcinoma led by multidisciplinary expert teams. Recent contributions made by thoroughly understanding pathophysiological, histological and molecular pathways involved in malignant alteration of adrenal cells by applying -OMICSs analyses of tumor samples have increased the scientific knowledge of ACC. However, only the integration of multi-center randomized, clinical, basic and genetic research results can accomplish comprehensive realization of victorious triumph against adrenocortical carcinoma.

## Figures and Tables

**Table 1:** Diagnostic (hormonal) work-up of suspected adrenal malignancies (modified according to 2020 guidelines [42]).

Indication	Assays	Specific Question
All adrenal masses with no overt Cushing (clinically)	1 mg dexamethasone suppression test	Exclusion of glucocorticoid excess
Adrenal masses with clinical signs of Cushing or pathological 1 mg dexamethasone test	1 mg dexamethasone suppression test	Characterization of glucocorticoid excess
	Free cortisol in 24-h urine	
	Basal ACTH (plasma)	
Any adrenal mass suspected to be an ACC	DHEA-S	Sex steroids precursors excess
	17-OH progesterone	
	Androstenedione	
	Testosterone (only in women)	
	17-beta-oestradiol (only in men and postmenopausal women)	
	11-deoxycortisol (if available)	
Any adrenal masses with hypertension and/or hypokalemia	Potassium	Mineralocorticoid excess
	Aldosterone/renin ratio	

17-OH, 17-hydroxy; ACC, adrenocortical carcinoma; ACTH, adrenocorticotropic hormone; DHEA-S, dehydroepiandrosterone sulfate.

**Table 2:** Review of novel immunohistochemically analyzed markers of adrenocortical carcinoma.

Marker	Definition/Role	Clinical Significance/Result	Number of Patients with Adrenocortical Carcinoma	Ref.
Metallothionein protein (MT) Minichromosome maintenance protein-2 (MCM2)	MT: scavengers of intracellular reactive oxygen species; overexpressed in various human tumors; MCM2: involved in the initiation of eukaryotic genome replication	-MT: no correlation with stage IV carcinoma -MCM2: positive correlation with Weiss revisited score, mitotic rate on histology, stage IV carcinoma	14	[85]
Minichromosome maintenance protein complex MCM-3, 5, 7	Replication-licensing proteins; increased levels of MCM are observed in dysplastic and neoplastic cells	-higher levels in ACC of MCM-3, MCM-7, but not MCM-5; -proliferative and diagnostic markers in discerning benign and malignant adrenocortical tumors.	3	[86]
Programmed death ligand (PD-L1 and 2)	Regulation of immune response; highly expressed in several cancers	-all tumor specimens were negative for PD-L1 expression; -PD-L2 is expressed commonly in adrenocortical adenomas samples	14; 34	[87,88]
Sterol-O-acyl transferase 1 (SOAT1)	Involved in cholesterol esterification and lipid droplet formation; SOAT1 inhibition leads to impaired steroidogenesis and cell viability in ACC	-37.5% of the ACCs demonstrated a strong SOAT1 protein expression (score > 2) -Strong SOAT1 protein expression correlated with features of high aggressiveness in ACC -SOAT1 expression was not correlated with recurrence-free survival, progression-free survival and disease-specific survival in ACC patients with mitotane monotherapy	112; 231	[89,90]
Somatostatin receptors (SSTRs)	Expressed in both normal tissues and solid tumors; part of distinct signaling cascades	-ACC can express SSTRs; SSTRs-based peptide receptor radionuclide therapy may represent a potential treatment opportunity for a minority of patients with advanced ACC	19	[91]
Chemokine receptor (CXCR 4 and 7)	Chemokine receptors have a negative impact on tumor progression in several human cancers	-High expression of CXCR4 and CXCR7 in both healthy and malignant adrenal tissue; strong membrane expression of CXCR4 and CXCR7 in 50% of ACC; -strong cytoplasmic CXCR4 staining was more frequent in metastases compared to primaries and local recurrences; -CXCR4 staining positively and CXCR7 negatively correlated with Ki67.	187	[92]
LH/CGR	Luteinizing hormone and/or chorionic gonadotropin (LH/CG) exert direct actions on the adrenal cortex and are involved in the adrenal pathology	-positive in the whole cytoplasm, but weak or absent in cell membranes; the loss of membrane localization of LH/CGR in adrenocortical cancer suggests the alteration of receptors’ function.	5	[93]
Fascin-1 (FSCN1) and FOXM1	Epithelial–mesenchymal transition (EMT) related genes	-FSCN1 and FOXM1 over-expression in ACC; -novel independent prognostic markers in ACC; -potential therapeutic target to block tumor spread	37; 51	[94,95]
Topoisomerase II alpha (TOP2A); thymidylate synthase (TS)	Prognostic parameters in several tumors and also predictors of efficacy of anthracyclines, topoisomerase inhibitors and fluoropirimidines	-TOP2A expression was associated with better after EDP-M (etoposide, doxorubicin and cisplatin plus mitotane) -TOP2A and TS were neither prognostic nor predictive of mitotane efficacy in ACC patients	39	[96]
Insulin like growth factor 2 (IGF2) IGF1 receptor (IGF1R)	Main pathway in ACC tumorigenesis	-in addition to IGF2 and IGF1R, ACC express IGF2R, IRA and several IGFBPs, suggesting that the interplay between the different components of the IGF pathway in ACC could be more complex than previously considered -IGF1 overexpression was associated with SLC12A7 overexpression and non-functional, early-stage and larger tumors	17; 33	[97,98]
CD276-(B7-H3)	Inhibitory role in adaptive immunity; in malignant tissues, B7-H3 is an immune checkpoint molecule	-positive expression on the cell membrane and in the cytoplasm of cancer cells or tumor-associated vascular cells -vascular expression of CD276 associated with local aggression	48	[99]
c-myc	Proto-oncogene	-strong cytoplasmic c-myc expression and weak nuclear expression in ACC associated with malignancy and shorter survival	31	[100]
Phosphorylated mTOR	Part of signaling pathway	-p-mTOR expression in 32% cases, with a moderate or strong cytoplasmic reactivity -p-mTOR was also negative in tumors with high Weiss Score,	58	[101]
Pituitary-tumor transforming gene (PTTG1)	Modulate cancer invasiveness and response to therapy	-increased nuclear protein expression of PTTG1 in ACC -PTTG1 correlated with Ki-67	20; 14	[102]
Glypicin-3 (GPC-3)	Role in the control of cell division and growth regulation; role in distinguishing hepatic lesions	-GPC-3 positivity rare in ACC, but possible, especially of extra-adrenal ACC	1; 2	[103,104]
E-/P-/N-cadherins, MMP-2/-9 and caveolin-1 ZEB-1/-2, Slug Oct3/4, LIN28, SOX2, SO17, NANOG, CD133, nestin	Epithelial–mesenchymal transition (EMT)-associated markers (E-/P-/N-cadherins, MMP-2/-9 and caveolin-1) Downstream transcriptional regulators of EMT-related signaling pathways (ZEB-1/-2, Slug) Stem cell factors (Oct3/4, LIN28, SOX2, SO17, NANOG, CD133, nestin) Markers of adrenocortical origin/tumorigenesis (SF-1, β-catenin, p53)	ACC with sarcomatous areas: -SF-1 and E-/P-/N-cadherins positive only in the epithelial component of all cases, whereas the nonepithelial components were mainly enriched for nestin, ZEB-1, and MMP-2/-9 -β-Catenin demonstrated an aberrant nuclear localization in the sarcomatoid component whereas p53 was strongly positive in the nonepithelial constituent	6	[105]
Livin/BIRC7	Member of the inhibitors of apoptosis proteins family, which are involved in tumor development through the inhibition of caspases	-over-expressed in ACC, localized in both cytoplasm and nuclei.-the ratio between cytoplasmic and nuclear staining was significantly higher in ACC than in ACA	192	[106]
Retinoic acid receptor responder 2 (RARRES2)	An immune-dependent tumor suppressor	-compared to normal adrenocortical tissues, expression was significantly lower in benign tumors, and even lower in ACC samples.	19	[107]
Adiponectin receptors	Adiponectin: involved in regulating glucose levels as well as fatty acid breakdown	-the expression of Adipo R1 and R2 receptors was associated with ACC diagnosis	20	[108]
Stathmin1 (STMN1)	Cytosolic protein involved in microtubule dynamics; implicated in carcinogenesis and aggressive behavior in multiple malignancies	-significantly higher expression of STMN1 protein in ACC compared with normal and benign tissues	13	[109]
MCT1, MCT2, MCT4, CD147, CD44, GLUT1 CAIX	MCT1 and MCT4 mediate monocarboxylate efflux from cells, while MCT2 is involved in monocarboxylate uptake; these transporters require co-expression with chaperones for proper plasma membrane localization and activity; the main chaperone is CD147; CD44 has also been recently described as a MCT chaperone	-increased membranous expression of MCT4, GLUT1 and CAIX in ACC -MCT1, GLUT1 and CAIX expressions associated with poor prognostic variables -MCT2 membranous expression was associated with favorable prognostic parameters. -cytoplasmic expression of CD147 was identified as an independent predictor of longer overall survival -cytoplasmic expression of CAIX as an independent predictor of longer disease-free survival.	78	[110]
VAV2	VAV2 -guanine nucleotide exchange factor; oncogene	-VAV2 expression correlated with Ki-67 index and progression free survival and overall survival	171	[111]
Cytochrome P450 genes	P450 overexpression potentiates adrenocortical carcinoma chemoresistance.	-analysis confirmed protein overexpression	29	[112]
Steroidogenic acute regulatory protein (StAR), CYP11B1, CYP11B2, YP17A1	Key proteins involved in the steroidogenesis cascade	-CYP11B1, StAR and CYP17A1 expression was lower in ACC; ACC presented co-staining cells for CYP11B1 and CYP11B2; CYP11B1 had the most discriminative power to distinguish ACC from ACA with a sensitivity of 100%, specificity of 92%	14	[49]
Progesterone receptor	Protein activated by the steroid hormone progesterone	-in 50% samples, IHC (immunohistochemistry) revealed a weak expression of progesterone receptor	8	[113]
D2-40; CD-31	D2-40 antibody for lymph vessels CD-31 antibody for blood vessels	-D2-40 expression lower in ACC and correlated positively with the expression of StAR; -CD31 expression higher in ACC	15	[114]
Inhibin, D2-40, synaptophysin	Inhibin downregulates FSH (follicle-stimulating hormone) synthesis and secretion; D2-40 may be a useful marker for distinguishing primary adrenal cortical neoplasms from both metastatic renal cell carcinoma and pheochromocytoma; Synaptophysin: major synaptic vesicle protein	-patients with any negative staining had shorter cancer-specific survival than ones with positive staining -negative staining for inhibin, D2-40 and synaptophysin and Ki-67 expression ≥7% were associated with poorer prognosis	30	[115]
Excision repair cross complementing group 1 (ERCC1)	Role in the repair of platinum-induced DNA damage	-high ERCC1 expression was observed in 66% of ACC samples	146	[116]
Sphingosine kinase 1 (SphK1)	Oncogene	-over-expression of SphK1 protein in the carcinomas compared with adenomas	24	[117]
N-cadherin	Aberrant expression of N-cadherin plays important role in ACC tumorigenesis	-N-cadherin downregulation was observed in 100 % of ACC	24; 15	[25,118]
Telomerase reverse transcriptase (TERT)	Catalytic subunit of the telomerase complex	-telomerase nuclear expression was present in 26.6% of ACC and in 45.5% of non-functioning adenomas	15	[118]
Isocitrate dehydrogenase (IDH) R132H mutation	Metabolic enzyme, ubiquitous in all cells; mutations of IDH play a prognostic or predictive role in several neoplasms	-positive IDH1 R132H staining correlated with a better prognosis among patients with ACC; it did not distinguish between local and metastasized tumors.	33	[119]
Indoleamine 2,3-dioxygenase 1 (IDO-1)	An immune checkpoint molecule	-IDO-1 is expressed in a majority of ACC samples; its expression in tumor tissue is associated with PD-L2 expression, and expression in stroma is associated with CD8+ cell infiltration.	32	[120]

## Appendix A

**Table A1:** Diagnostic criteria/scoring in differentiating malignant from benign adrenocortical lesions [42,121,123,234].

Weiss Criteria ≥ 3 Criteria	Wieneke Criteria * ≥ 4 Criteria	Lin–Weiss–Bisceglia System **:
1. High nuclear grade (III or IV) ^†^ 2. Mitotic rate greater than 5 per 50 high-power fields 3. Presence of atypical mitoses 4. Clear lipid-rich cells comprising less than 25% of the tumor 5. >33% diffuse architecture 6. necrosis 7. Invasion of venous structures 8. Invasion of sinusoidal structures 9. Invasion of the capsule	1. Tumor weight >400 g 2. Tumor size >10.5 cm 3. Extension into periadrenal soft tissues and/or adjacent organs 4. Invasion into vena cava 5. Venous invasion 6. Capsular invasion 7. Presence of tumor necrosis 8. >15 mitoses per 20 HPF 9. Presence of atypical mitotic figures	Major criteria 1. Mitotic count >5 per 50 high- power fields 2.Atypical mitoses 3. Venous invasion Minor criteria 1. Size >10 cm and/or weight >200 g 2. Necrosis 3. Sinusoidal invasion 4. Capsular invasion

Each criterion is scored 0 when absent and 1 when present in the tumor; * Modified Weiss scoring system: calculate: 2× mitotic rate criterion + 2× clear cytoplasm criterion + abnormal mitoses + necrosis + capsular invasion (for a total of 7 possible points; a score of 3 or more suggests malignancy); ** the presence of one major criterion indicates malignancy, one to four minor criteria present indicate uncertain malignant potential, and the absence of all major and minor criteria is indicative of benign biological behavior; ^†^ Fuhrman criteria (nucleus): grade 1 (round nuclei, homogenous, small size, no nucleoli), grade 2 (nuclei slightly irregular, more voluminous, conspicuous nucleoli at ×400), grade 3 (irregular nuclei, voluminous nucleoli at ×100), grade 4 (idem grade 3 with monstrous cells with very irregular nucleus.
